# Risk factors for postoperative acute kidney injury after radical cystectomy for bladder cancer in the era of ERAS protocols: A retrospective observational study

**DOI:** 10.1371/journal.pone.0309549

**Published:** 2024-10-15

**Authors:** Mathieu Marques, Marie Tezier, Maxime Tourret, Laure Cazenave, Clément Brun, Lam Nguyen Duong, Sylvie Cambon, Camille Pouliquen, Florence Ettori, Antoine Sannini, Frédéric Gonzalez, Magali Bisbal, Laurent Chow-Chine, Luca Servan, Jean Manuel de Guibert, Marion Faucher, Djamel Mokart

**Affiliations:** 1 Service d’Anesthésie-Réanimation, Institut Paoli Calmette, Marseille, France; 2 Service d’Anesthésie-Réanimation, Hôpital Louis Pradel, Hospices Civils de Lyon, France; Stanford University School of Medicine, UNITED STATES OF AMERICA

## Abstract

**Background:**

Radical cystectomy (RC) is a major surgery associated with a high morbidity rate. Perioperative fluid management according to enhanced recovery after surgery (ERAS) protocols aims to maintain patients in an optimal euvolemic state while exposing them to acute kidney injury (AKI) in the event of hypovolemia. Postoperative AKI is associated with severe morbidity and mortality. Our main objective was to determine the association between perioperative variables, including some component of ERAS protocols, and occurrence of postoperative AKI within the first 30 days following RC in patients presenting bladder cancer. Our secondary objective was to evaluate the association between a postoperative AKI and the occurrence or worsening of a chronic kidney disease (CKD) within the 2 years following RC.

**Methods:**

We conducted a retrospective observational study in a referral cancer center in France on 122 patients who underwent an elective RC for bladder cancer from 01/02/2015 to 30/09/2019. The primary endpoint was occurrence of AKI between surgery and day 30. The secondary endpoint was survival without occurrence or worsening of a postoperative CKD. AKI and CKD were defined by KDIGO (Kidney Disease: Improving Global Outcomes) classification. Logistic regression analyse was used to determine independent factors associated with postoperative AKI. Fine and Gray model was used to determine independent factors associated with postoperative CKD.

**Results:**

The incidence of postoperative AKI was 58,2% (n = 71). Multivariate analysis showed 5 factors independently associated with postoperative AKI: intraoperative restrictive vascular filling < 5ml/kg/h (OR = 4.39, 95%CI (1.05–18.39), p = 0.043), postoperative sepsis (OR = 4.61, 95%CI (1.05–20.28), p = 0.043), female sex (OR = 0.11, 95%CI (0.02–0.73), p = 0.022), score SOFA (Sequential Organ Failure Assessment) at day 1 (OR = 2.19, 95%CI (1.15–4.19), p = 0.018) and delta serum creatinine D1 (OR = 1.06, 95%CI (1.02–1.11), p = 0.006). During the entire follow-up, occurrence or worsening of CKD was diagnosed in 36 (29.5%). A postoperative, AKI was strongly associated with occurrence or worsening of a CKD within the 2 years following RC even after adjustment for confounding factors (sHR = 2.247, 95%CI [1.051–4.806, p = 0.037]).

**Conclusion:**

A restrictive intraoperative vascular filling < 5ml/kg/h was strongly and independently associated with the occurrence of postoperative AKI after RC in cancer bladder patients. In this context, postoperative AKI was strongly associated with the occurrence or worsening of CKD within the 2 years following RC. A personalized perioperative fluid management strategy needs to be evaluated in these high-risk patients.

## Background

Surgical radical cystectomy (RC) remains a crucial step in the treatment of bladder cancer. However, RC is a major surgical procedure associated with a high morbidity rate, even when ERAS programs are applied [1]. A meta-analysis of randomized controlled trials has demonstrated a reduction in postoperative morbidity and length of hospital stay since the introduction of enhanced recovery after surgery (ERAS) protocols. [2]. Perioperative fluid management according to the ERAS protocol aims to maintain the patient in the optimal euvolemic status while avoiding the critical effects of fluid overload. From an anesthetic point of view, ERAS guidelines induced changes of practice and are now largely validated in major surgery [3]. We have recently shown that the implementation of ERAS protocols, particularly in colorectal and abdominal gynecological oncological surgery, reduces minor postoperative complications and even mortality in elderly subjects [4–6]. In this context, perioperative hydration management protocols aimed at neutral fluid balance have been shown to reduce the incidence of major and gastrointestinal complications in patients undergoing major abdominal surgery [7]. Furthermore, the implementation of restrictive hydration strategies has been associated with lower mortality rates and reduced complications following major abdominal surgery, as demonstrated in recent literature [8]. However, there is a persistent concern that restricted hydration could lead to hypovolemia, which could result in renal dysfunction. [9]. Perioperative urine output is commonly used as an indicator of renal function, and additional fluid boluses are often administered to overcome oliguria, although evidence for their efficacy is limited [8]. Following RC, a wide range of postoperative complications have been described including gastro-intestinal, urinary and renal complications [10]. The incidence of acute kidney injury (AKI) after RC is common. It ranges between 5% and 40% according to previous reports, depending on the definition [11–14]. In this situation, usually found predictive factors are comorbidities, high-grade postoperative complications, sex (male), operative time, neoadjuvant chemotherapy [11–14]. Postoperative AKI is also associated with severe morbidity and mortality [15, 16]. Of note, restrictive intraoperative fluid administration has been associated with an increased risk of developing AKI, particularly in elderly patients, those receiving antihypertensive drugs and those undergoing prolonged surgical procedures [17]. It has been shown that patients who partially recovered from an episode of AKI are at higher risk of long-term mortality [16, 18]. Despite the standardization of surgical techniques and the introduction of robotic laparoscopy, RC remains a complex procedure with multiple risks of postoperative complications including AKI [1, 13, 19]. Interestingly, those who completely recovered from an episode of AKI are more likely to develop incident chronic kidney disease (CKD) [20]. CKD after RC occurs in 25% to 50% of cases. In this situation, the main associated factors include preoperative hydronephrosis, pyelonephritis, nephrotoxic chemotherapy and baseline hypertension [21–23]. Our main objective was to determine the association between perioperative variables, including some component of ERAS protocols such as intraoperative vascular filling, and occurrence of postoperative AKI within the first 30 days following RC in patients presenting bladder cancer. Our secondary objective was to evaluate the association between a postoperative AKI and the occurrence or worsening of a CKD within the 2 years following RC.

Briefly, in this study we will first evaluate the incidence of postoperative AKI after RC, secondly the risk factors associated with it and finally we will assess the impact of AKI on the occurrence or aggravation of CKD.

### Patients

#### Study design

We conducted a retrospective observational study in a referral cancer center in France (Institut Paoli-Calmettes, Marseille). The study was approved by our IRB which waived the need of informed consent (CYST-IRAAC-IPC 2019–047). In our institution, most of patients presenting with high grade non-muscle invasive (NMIBC) or invasive (MIBC) bladder cancer are treated with laparotomy or robotic-assisted RC with pelvic lymphadenectomy [24]. Details on preoperative chemotherapy (Table 1), type of surgery (Table 2) and TNM stage (Table 3) are provided in the results section. All patients undergoing an elective RC for bladder cancer from 01/02/2015 to 30/09/2019 at Paoli-Calmettes Institute were included in the study. Data were collected until 06/2020. Exclusion criteria were as follows: age < 18 years, ASA score >3, partial cystectomy, all reinterventions after partial cystectomies and RC performed for locally advanced gynecologic cancer, frozen pelvis.

**Table 1:** Pre-operative characteristics.

	All patients (n = 122)	No AKI (n = 51)	AKI (n = 71)	p value
**Age** (years)	71 [63–76]	71.00 [61.50–76.00]	71.00 [64.50–76.00]	0.493
**Sex** (%)				0.002
Male	90 (73.8)	30 (58.8)	60 (84.5)	
Female	32 (26.2)	21 (41.2)	11 (15.5)	
Body mass index (kg/m^2^)	24.9 [22.6–37.7]	24.20 [21.50–26.50]	25.10 [23.40–28.25]	0.038
**ASA score** (%)				0.183
ASA 1	4 (3.3)	3 (5.9)	1 (1.4)	
ASA 2	71 (58.2)	32 (62.7)	39 (54.9)	
ASA 3	47 (38.5)	16 (31.4)	31 (43.7)	
**Antecedents**				
Charlson Score	5 [4–6]	5.00 [4.00–6.00]	5.00 [4.00–6.00]	0.246
Hypertension	61 (50)	23 (45.1)	38 (53.5)	0.463
RAAS inhibitors	40 (32.8)	14 (27.5)	26 (36.6)	0.332
Diabetes	16 (13.1)	6 (11.8)	10 (14.1)	0.918
Smokers	38 (31.1)	18 (36.7)	20 (28.2)	0.328
Peripheral vascular disease	12 (9.8)	4 (7.8)	8 (11.3)	0.759
Myocardial Infarction	16 (13.1)	4 (7.8)	12 (16.9)	0.18
**Preoperative biology**				
Hemoglobin (g/dL)	11.9 [10.7–12.9]	12.00 [10.85–12.9]	11.80 [10.75–12.70]	0.801
Serum Albumin level (g/L)	40 [34–42.5]	38.50 [34.25–41.00]	41.00 [34.00–43.00]	0.354
SCr (μmol/L)	97.5 [79–125]	86.00 [77.50–121.50]	101.00 [82.00–125.00]	0.268
GFR (ml/min/1,73m^2^)	65 [48–81]	65.00 [47.75–86.00]	64.50 [49.75–73.75]	0.688
GFR< 60 ml/min/1,73m^2^	66 (54.1)	26 (51.0)	40 (56.3)	0.585
**Preoperative chemotherapy**				
MVAC	45 (39.5)	15 (29.4)	30 (42.3)	0.147
GemCis	14 (11.5)	6 (11.8)	8 (11.3)	0.932
Cisplatin	60 (49.2)	22 (43.1)	38 (53.5)	0.258
MTX	45 (36.9)	15 (29.4)	30 (42.3)	0.147
**Preoperative urologic status**				
Single kidney	5 (4.1)	2 (3.9)	3 (4.2)	0.933
Preoperative ureteral double J stent	29 (23.8)	20 (39.2)	9 (12.7)	0.001

Data are presented in median [quartiles] and in terms of population (percentages).

AKI, Acute Kidney Injury; BMI, Body Mass Index; ASA, American Society of Anesthesiologist; ERAS, Early Recovered After Surgery; RAAS, Renin–angiotensin–aldosterone system; SCr, Serum Creatinine level; GFR, Glomerular Filtration Rate; MVAC, Methotrexate-Vinblastine-Doxorubicin-Cisplatin; GEMCis, Gemcitabin-Cisplatin; MTX, Methotrexate.

**Table 2:** Intraoperative characteristics, univariate analysis.

	No AKI (n = 51)	AKI (n = 71)	p value
**Surgical technique**			
Laparotomy	33 (64.7)	32 (45.1)	0.032
Robotic assisted surgery	25 (49)	49 (69)	0.026
Mixed procedure	7 (9.8)	10 (14.1)	0.955
**RC and urine diversion**			
Ileal conduit	26 (51.0)	31 (43.7)	0.465
Neobladder construction	24 (47)	38 (53.5)	0.481
Ureterocutaneostomy	1 (2)	2 (2.8)	0.571
**Duration of procedure**			
Anesthesia (min)	486.00 [424.50–568.50]	575.00 [485.50–618.00]	0.001
Surgery (min)	386.00 [332.50–469.00]	464.00 [376.00–512.50]	0.002
Laparoscopy (min)	0.00 [0.00–390.00]	356.50 [0.00–453.00]	0.017
**Intraoperative hemodynamic status**			
Postoperative Lactate (mmol/L)	2.40 [1.73–2.90]	2.30 [1.70–3.00]	0.869
Norepinephrine	4 (7.8)	9 (12.7)	0.554
Crystalloids (ml/kg/h)	5.55 [4.43–7.97]	4.80 [3.55–6.45]	0.018
Total intra vascular filling (ml/kg/h)	5.55 [4.50–8.28]	4.85 [3.55–6.45]	0.016
Restrictive IV filling < 5 ml/kg/h	24 (47.1)	47 (66.2)	0.035
Blood transfusion	7 (13.7)	7 (9.9)	0.709
Blood loss (ml)	400.00 [250.00–550.00]	400.00 [275.00–600.00]	0.979
Fluid Balance (ml)	1836.00 [1403.00–2752.00]	1912.00 [1349.50–2565.75]	0.981
Cardiac output monitoring	4 (7.8)	6 (8.6)	1
End-surgery body temperature	36.60 [36.15–36.90]	36.80 [36.48–37.10]	0.069
**Drugs used intraoperatively**			
NSAIDs	28 (53.8)	33 (47.1)	0.583
Aminoglycosides	2 (3.9)	7 (9.9)	0.302
Morphine IV	19 (37.3)	19 (27.1)	0.321
Lidocaine	28 (54.9)	50 (71.4)	0.083
Lidocaine (mg)	70.00 [0.00–513.00]	535 [0.00–734.25]	0.017
Ketamine	48 (94.1)	66 (94.3)	1
Ketamine (mg)	80.00 [60.00–90.00]	90.00 [72.50–100.00]	0.005
**Type of loco regional anesthesia**			
Epidural Anesthesia	10 (19.6)	22 (31.4)	0.210
Intrathecal morphine	2 (3.9)	1 (1.4)	0.176
Parietal Lidocaine Infusion	15 (29.4)	22 (31.4)	0.844

Data are presented in median [quartiles] and in terms of population (percentages).

AKI, Acute Kidney Injury; IV, Intravenous; GFR, Glomerular Filtration Rate; NSAIDs, Non-Steroidal Anti-Inflammatory Drugs.

**Table 3:** Postoperative characteristics.

	No AKI (n = 51)	AKI (n = 71)	p value
**SAPS II**	21.00 [16.50–27.50]	26.00 [21.50–31.50]	0.001
**SOFA**			
Day 1	1.00 [1.00–2.00]	2.00 [2.00–3.00]	0.001
Day 3	0.00 [0.00–1.00]	0.00 [0.00–1.00]	0.933
**Clavien-Dindo classification**			0.060
No or low-grade complications	41 (80.4)	46 (64.8)	
High-grade complications	10 (19.6)	25 (35.2)	
**Complications**			
Ileus	17 (33.3)	32 (45.1)	0.261
Urinary tract infection	16 (31.4)	35 (49.3)	0.063
Sepsis	18 (35.3)	42 (59.2)	0.011
Blood transfusion	9 (17.6)	24 (33.8)	0.076
Revision surgery	4 (7.8)	8 (11.3)	0.759
Radiological Intervention	4 (7.8)	10 (14.0)	0.391
Kidney Dialysis	0 (0)	3 (4.2)	0.264
Intensive care Unit	0 (0)	11(15.5)	0.15
**Postoperative biology at Day 1**			
Lactate (mmol/L)	2.40 [1.73–2.90]	2.30 [1.70–3.00]	0.869
SCr (mmol/L)	96 [74–123]	126 [106–161]	<0.001
Na+ (mmol/L)	136.00 [134.00–138.00]	137.00 [135.00–138.00]	0.274
K+ (mmol/L)	4.50 [4.25–4.80]	4.80 [4.50–5.65]	0.002
HCO3- (mmol/L)	23.00 [21.00–25.00]	22.00 [20.00–24.50]	0.346
Hemoglobin (g/dL)	10.30 [9.20–11.20]	10.00 [9.40–11.30]	0.936
CPK (UI/L)	511.00 [233.00–1456.00]	1074.00 [382.00–2310.00]	0.122
**Postoperative biology at Day 3**			
SCr (μmol/L)	76 [60–102]	96[76–114]	0.001
Na+ (mmol/L)	136.00 [133.00–138.00]	135.00 [134.00–138.00]	0.733
K+ (mmol/L)	3.90 [3.70–4.10]	4.00 [3.80–4.30]	0.063
HCO3- (mmol/L)	26.00 [24.00–28.00]	26.00 [24.00–28.00]	0.766
Hemoglobin (g/dL)	9.70 [9.00–10.55]	9.40 [9.00–10.75]	0.864
**Delta SCr**			
Day 1 –Preoperative (μmol/L)	-4 [-13; -10]	26 [9; 44]	<0.001
Day 3 –Preoperative (μmol/L)	-19 [-28; -7]	-4 [-22; 5.5]	<0.001
**ERAS Protocol**			
No ERAS	19 (37.3)	19 (26.8)	0.288
Compliance ≥ 75%	34 (66.7)	52 (73.2)	0.432
Compliance <75%	17 (33.3)	19 (26.8)	0.432
**AJCC 8**^**th**^ **edition pTNM staging**			
**0is**	4 (7.8)	8 (11.3)	0.554
**0a**	7 (13.7)	18 (25.4)	0.116
**I**	4 (7.8)	8 (11.3)	0.554
**II**	8 (15.6)	6 (8.5)	0.264
**IIIa**	17 (33.3)	19 ((26.8)	0.551
**IV**	11 (21.6)	12 (16.9)	0.353
**Length of stay (days)**			
Intermediate care unit	5.00 [3.00–6.00]	5.00 [3.50–7.00]	0.186
Hospital	11.00 [9.00–13.00]	12.00 [10.00–14.00]	0.108
**Re-hospitalization**	8 (15.7)	16 (22.5)	0.688

Data are presented in median [quartiles] and in terms of population (percentages).

AKI, Acute Kidney Injury; SAPS, Simplified Acute Physiology Score; SOFA, Sequential Organ Failure Assessment; Scr, Serum Creatinine level; Na+, Natremia; K+, Kalaemia; HCO3-, Sodium Bicarbonate; CPK, Creatinine Phospho Kinase; Delta SCr, Difference between serum creatinine level; ERAS, Early Recovery After Surgery. AJCC 8^th^ edition pTNM staging, American Joint Committee on Cancer, 8th edition pathological Tumour Node Metastasis staging

#### Anesthesia and perioperative analgesia

From January 2018 (second period), all patients treated for a bladder cancer by RC benefited from a specifically designed urological ERAS protocol [25] (S1 File). Nevertheless, some patients who underwent RC in the first period could be treated according to components of the ERAS urological protocols even though these were not yet in place at that time. Indeed, some of ERAS protocol components are common between the different oncological major surgeries (e.g., intraoperative vascular filling, intraoperative protective ventilation, intraoperative intravenous lidocaine…). For all patients, anesthesia and analgesia protocols were standardized (S2 File). For most patients, protective ventilation during surgery was started immediately from intubation time to the end of the surgical procedure and included low tidal volume objective (6–8 ml/kg of ideal weight), PEEP ≥ 5 cmH2O and recruitment maneuvers [26]. During the second period and for most patients, fluid administration was performed according to the ERAS protocol objective with a restrictive intraoperative vascular filling (<5ml/kg/h) [25]. During the first period fluid administration was let at anesthesiologist discretion. For post-operative nausea and vomiting prophylaxis, 8 mg of intravenous dexamethasone and 1.25 mg of intravenous droperidol were administrated intraoperatively. Four milligrams of ondansetron were administered if Apfel score was ≥ 3 [27]. Placement of an analgesic epidural catheter before the induction of GA was possible at the anesthetist’s discretion, for patients undergoing laparotomy. Intra-operative intravenous analgesia was administered with a 0.5 mg/kg bolus of ketamine at induction. Lidocaine administration was protocolized: a bolus of 1 mg/kg of ideal weight was delivered during induction of anesthesia and followed by 1.5 mg/kg/h during surgery. It was stopped one hour before the end of the surgery and analgesia was completed most often with a Transversus Abdominis Plane block (TAP block) or surgical parietal infiltration of ropivacaine 7.5% for laparotomy and a surgical infiltration of trocar holes for robot assisted surgery. At the end of surgery, a multimodal approach of analgesia was employed with an opioid sparing approach (with anti-inflammatory drugs, ketamine, co analgesic (paracetamol, nefopam) and patient-controlled intravenous analgesia (PCA) pump (morphine 1 mg/ml, bolus: 1.5 ml, lockout time: 7–10 min, maximum cumulative dose for 4h: 20–30 mg) for 48h. Other modalities of analgesia (like spinal analgesia or thoracic epidural analgesia for 48–72 post-operative hours) were employed when the anesthetists decided it was necessary. Epidural analgesia was maintained during the 3 post-operative days with naropeine 2% (10 mg/h) and sufentanil (1 μg/h). Spinal analgesia was performed immediately before the induction of anesthesia using morphine 1% (0.3mg). All patients were systematically admitted to the intermediate care unit (IMC) for standardized perioperative care [28].

#### Data collection and definitions

Patient characteristics, disease status, intraoperative variables, and outcomes were summarized through descriptive statistics in Tables 1–3. Demographic data, comorbidities such as severe chronic kidney disease (CKD) [29], Charlson Comorbidity Index Score [30], Karnofsky Index [31] and American Society of Anesthesiologists Physical Status Classification (ASA score) [32] were recorded using our hospital information system (Hopital Manager, Softway Medical, France). Important perioperative parameters were prospectively gathered from our Anesthesia and ICU information system (Metavision, iMDsoft, Wakefield, U.S.A.). Postoperatively, important parameters such as SAPS II [33] and SOFA score [32] at Day 1 (D1) and Day 3 (D3) were systematically recorded as well as postoperative complications, sepsis, occurrence of AKI and CKD, changes in postoperative creatinine levels and compliance to ERAS protocol.

#### Definitions

The SOFA and SAPS II scores are two commonly used clinical scoring systems to assess the severity of illness and predict the outcome of patients admitted to the ICU. The SOFA score evaluates organ dysfunction in six different organ systems, including the respiratory, cardiovascular, hepatic, coagulation, renal, and neurological systems. Higher SOFA scores indicate a greater degree of organ dysfunction, and it is often used to monitor the progression of organ failure during a patient’s stay in the ICU. On the other hand, SAPS II is a scoring system that takes into account various physiological and clinical parameters, such as age, vital signs, and laboratory values, to predict the risk of mortality for ICU patients. A higher SAPS II score indicates a greater risk of mortality, and it is commonly used for risk stratification. Both scores provide valuable information for clinicians to assess the condition and prognosis of patients in the ICU. Complications within 30 days after surgery were identified and classified according to the Clavien-Dindo classification of surgical complications [34]. A severe complication was defined as a Clavien-Dindo classification Grade IIIa to IVb. Sepsis was defined according to Sepsis 3 by suspect infection (hyperleukocytosis or leukopenia, fever, positive bacteriological samples) and SOFA > = 2 [35]. AKI was defined by Kidney Disease Improving Global Outcomes (KDIGO) with the occurrence of 1 of the 3 items: 1) increase in serum creatinine by ≥ 26,5 μmol/L within 48 hours; 2) increase in serum creatinine ≥ 1,5-time baseline, which is known or presumed to have occurred within the prior seven days; 3) urine volume < 0,5 ml/kg/h during 6 hours, KDIGO score were also recorded [36]. In addition, we calculated the Delta SCr Day 1 and Delta SCr Day 3 (serum creatinine levels at Day 1 or Day 3 minus preoperative serum creatinine level). We also collected all the necessary parameters to evaluate ERAS protocol compliance according to the protocol as described in S1 File. For each patient, compliance to ERAS protocol was met if 18 of the 24 criteria (≥75%) were fulfilled. The 24 perioperative items of ERAS protocol are described in S3 File. The compliance rate for all patients was also assessed and expressed as a percentage. CKD was defined by a decreased kidney function (glomerular filtration rate [GFR]) <60 mL/min per 1·73 m²) for 3 months or more [37, 38].

All data were collected by 2 physicians (LC/MM), discordances were subsequently analyzed by a local adjudication committee. Finally, hospital mortality, IMC and hospital lengths of stay, hospital readmission and vital status, alive or death of any cause, at the end of the follow-up were also recorded.

### Main outcomes

#### Study endpoints

The primary endpoint of this study was occurrence of AKI between surgery and Day 30. The secondary endpoint was survival without occurrence or worsening of CKD within the 2 years following RC.

#### Follow up

Overall, patients were followed-up according to the cancer bladder guidelines [25]. In this context, the short-term follow-up was from the day of surgery until day 30. Long-term follow-up was from the day of surgery until 24 months after surgery. The patients were followed after hospital discharge using the hospital information system, which is used for administrative and medical purposes throughout every procedure, visit, laboratory examination, vital sign, and other data gathered during hospitalizations or outpatient visits. All data are compulsorily recorded along with the date and a unique identifier.

### Statistical analysis

All of the data are presented as rates (percentages) for the qualitative variables and as median [25th-75th percentiles] or mean [standard deviations (SD)] for the quantitative variables. Data were compared between two group of patients: occurrence of AKI within the first 30 days of the post-operative period (AKI group) or no AKI during this period (no AKI group). Comparisons between the 2 groups of patients were realized using the Mann-Whitney test for continuous variables and the Chi-Square or Fisher’s exact tests for categorical variables. All p values < 0.05 were considered to be statistically significant. We performed a logistic regression analyses to identify independent variables associated with the development of postoperative AKI, as measured by the estimated odds ratio (OR) and 95% confidence interval [95% CI]. Factors with significance or borderline significance (p< 0.1) in the univariate analyses and those related as pertinent factors in the literature were then included in a multivariable regression model with backward stepwise variable selection (S4 File). We chose 0.1 as the critical *p* value for entry into the model and 0.1 as the *p* value for removal. The required significance level was set at a p value < 0.05. The Hosmer-Lemeshow test was used to check goodness-of-fit of the selected logistic model.

In the second part, we analyzed the association of postoperative AKI with occurrence or worsening of a CKD within the 2 years following surgery. Since patients may die before occurrence or worsening of a CKD, we used a competing-risks analysis as described by Fine & Gray which accounts for the competing risk of death without CKD or death without worsening of a preexisting CKD [39]. CKD free survival was defined as the interval between the date of surgery and the date of CKD occurrence or worsening, or last follow-up or death from any cause. For this endpoint the follow-up period was censored at 24 months. Bivariable Fine and Gray model was used to evaluate the effect of postoperative AKI alone or with confounding factors on occurrence or worsening of a CKD (S4 File). Results were expressed as sub hazard ratios (sHR) and 95% confidence intervals [CIs]. Cumulative incidence curves were used to describe cumulative incidence of occurrence or worsening of a CKD and comparisons between groups were performed using Gray’s test [40]. Association between postoperative AKI and 2-year mortality was evaluated using log-rank test. All tests were two-sided, and p values lower than 0.05 were considered statistically significant. Statistical tests were conducted using the SPSS 13 software package (IBM, Armonk, NY, USA) and R software, version 3.4.3 (available on line at: https://www.rproject.org/).

## Results

### Characteristics of patients

**Fig 1:**
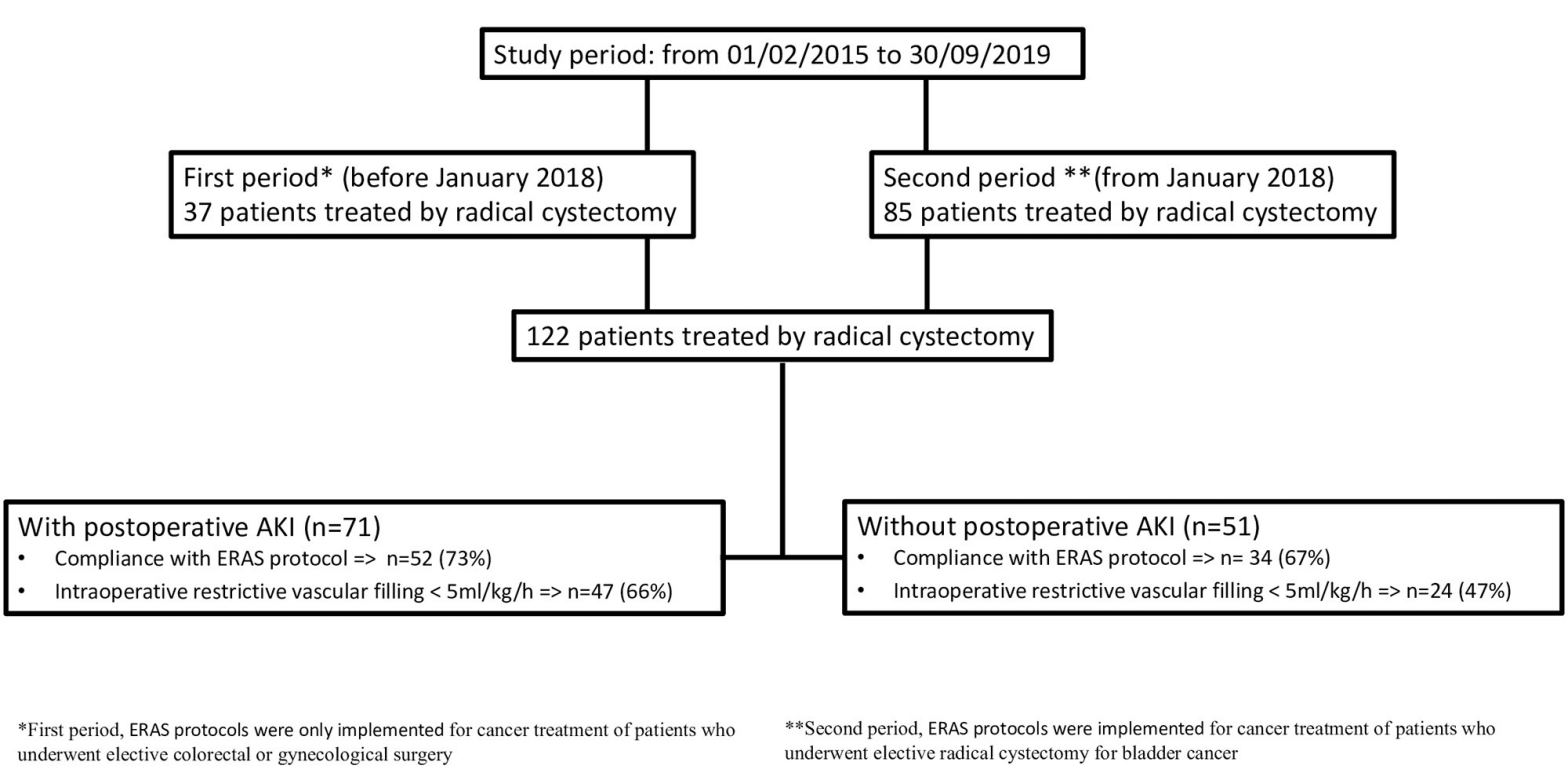
Study flowchart.

From February 2015 to September 2019, 122 patients were included in the study (Fig 1). No patients were excluded. Regarding the overall cohort (Table 1), 90 (73.8%) patients were male, the median age was 71 [63–76] years, the Charlson index 5 [4–6] and 47 (38.5%) patients had ASA score > 2 (Table 1). Prior to surgery, 45 (39.5%) patients had been treated with neoadjuvant MVAC (Methotrexate-Vinblastine-Doxorubicin-Cisplatin) chemotherapy and 14 (11.5%) with GemCis (Gemcitabine-Cisplatin) chemotherapy. During the intraoperative period (Table 2), the main surgical approach was robotic assisted surgery for 74 (60.7%) patients, laparotomy was realized for 65 (53.3%) of which 17 (13.9%) were treated with a mixed procedure combining the 2 procedures (robotic surgery followed by a conversion laparotomy). The most frequent surgical technique was orthotopic neobladder for 59 (48.3%) patients. Surgery duration was 421 minutes (346–501), norepinephrine was used in 13 patients (11%), total vascular filling was 5.1 ml/kg/h (3.8–7.5), blood loss 400 ml (250–600) and 14 patients received (11.4%) an intraoperative blood transfusion. The postoperative complication rate at day 30 was 80.3% (n = 98) of which 35 (28.6%) patients had Dindo-Clavien complications > grade II. At day 30, 71 (58.2%) patients developed an AKI, the mean time to onset of postoperative AKI was 4.5 (6) days. Twenty-four (20%) patients developed a postoperative AKI after day 1. According to KDIGO criteria: 52 were classified in a stage 1 (73.2%), 10 in a stage 2 (14.1) and 9 in a stage 3 (12.7%). Finally, 3 (2,4%) patients needed renal replacement therapy (RRT). By the 10th day of IMC admission, 57 of the 71 patients who had developed AKI (80%) had fully recovered from their AKI, and by the 30th day, 66 (93%) patients had fully recovered from their AKI.

During the study period, 86 (70%) patients received an ERAS protocol with a compliance rate ≥ 75%. There was no significant difference in the compliance with ERAS protocols between the AKI and non-AKI groups (52 (73.2%) vs 34 (66.7%), respectively; p = 0.432 and Table 3). However, during the first period of the study (n = 38), the compliance with ERAS protocols was lower than during the second period (n = 84) (13 (34%) vs 72 (87%), respectively; p<0.001) while the occurrence of AKI was comparable (20 (50%) vs 52 (62%), respectively; p = 0.288).

Postoperative sepsis was present in 60 (49%) patients, in addition 12 (10%) patients underwent surgical reoperation and 14 (11%) an interventional radiology procedure for which in 11 patients urine was diverted via a percutaneous nephrostomy tube, and in 3 patients percutaneous drainage of an intrabdominal collection was performed. Hospital length of stay was 11 [10–14] days; mortality at day 30 in hospital and at day 90 was zero.

By univariate analysis (Tables 1–3), the main factors associated with postoperative AKI were sex (p = 0.002), BMI (p = 0.038), preoperative double J stent (p = 0.001), robotic assisted surgery (p = 0.016), restrictive intraoperative vascular filling < 5ml/kg (p = 0.035), SAPSII score (p = 0.001), SOFA score on day 1 (p = 0.001), duration of surgery (0.002), duration of laparoscopy (p = 0.017, postoperative sepsis (p = 0.011), serum potassium on day 1 (0.002), delta serum creatinine D1 (p<0.001) and delta serum creatinine D3 (p<0.001). Neither the type of chemotherapy nor the pTNM stage was associated with the occurrence of postoperative AKI.

Multivariate analysis showed 5 factors independently associated with postoperative AKI: intraoperative restrictive vascular filling < 5ml/kg/h (OR = 4.39, 95%CI (1.05–18.39), p = 0.043), postoperative sepsis (OR = 4.61, 95%CI (1.05–20.28), p = 0.043), female sex (OR = 0.11, 95%CI (0.02–0.73), p = 0.022), SOFA score (Sequential Organ Failure Assessment) at day 1 (OR = 2.19, 95%CI (1.15–4.19), p = 0.018) and delta serum creatinine D1 (OR = 1.06, 95%CI (1.02–1.11), p = 0.006).

**Fig 2:**
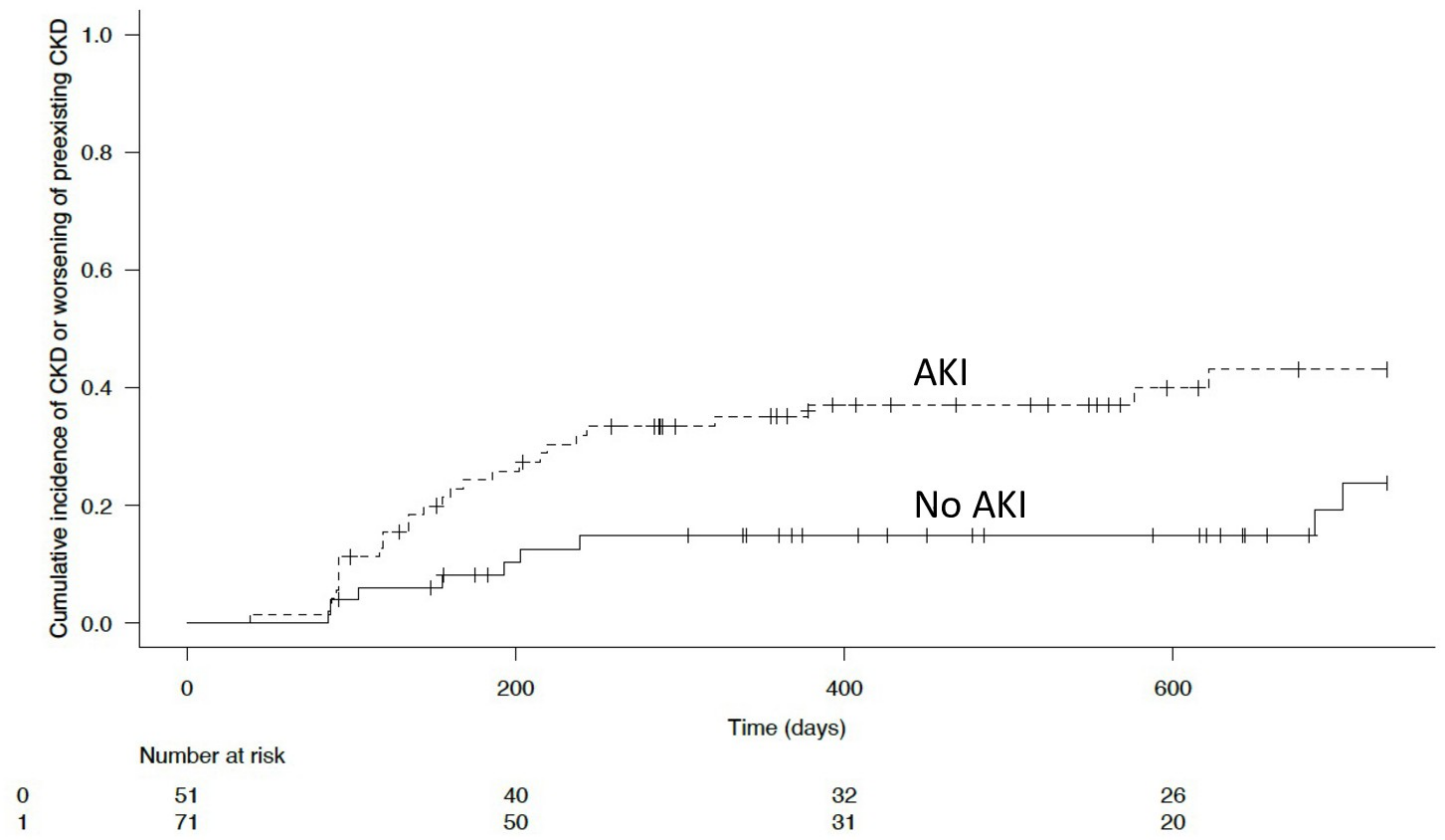
Cumulative incidence of CKD or worsening of preexisting CKD according to postoperative AKI.

The median follow-up from the time of surgery was 19 months (95% CI, 16.2–21.2 months), 23 (19%) patients died during the study period and 70 patients were lost to follow-up. Postoperative AKI was not associated with 2-year mortality, p = 0.959. During the entire follow-up, occurrence or worsening of CKD was diagnosed in 36 (29.5%; 95%CI, 21.3%-37.7%) patients, this diagnosis was nevertheless achieved in 18 patients (26%) lost to follow-up. Finally, a postoperative AKI was strongly associated with occurrence or worsening of a CKD within the 2 years following RC (sHR = 2.471, 95%CI [1.16–5.263, p = 0.019]). After adjustment for confounding factors (MVAC, GemCis, preoperative CKD, Charlson comorbidity index, ASA score, intraoperative restrictive fluid management, intraoperative vasopressors, ERAS protocol, robot assisted surgery, SOFA score at day 1,serum potassium level at day 1, serum potassium level at day 3, SAPS II score, postoperative complications = Clavien-Dindo stages and postoperative RRT) postoperative AKI was still associated with occurrence or worsening of a CKD (sHR = 2.247, 95%CI [1.051–4.806, p = 0.037]), S5 File. Of the 36 patients who developed CKD or worsened pre-existing CKD, 2 (5.5%) were treated with dialysis during the follow-up period. Cumulative incidence of occurrence or worsening of CKD according to postoperative AKI are showed Fig 2, p = 0.015.

## Discussion

This observational study included 122 patients who underwent RC for bladder cancer. The incidence of postoperative AKI was 58.2%, and factors independently associated with postoperative AKI were a postoperative increase in serum creatinine on day 1 compared with baseline, a high SOFA score on day 1, postoperative sepsis, male gender and intraoperative restrictive vascular filling < 5ml/kg/h. Postoperative AKI was independently associated with the occurrence or worsening of CKD within the 2 years following RC.

### Postoperative AKI

In our study, AKI incidence was higher as compared with recent studies. Indeed, in this context numerous studies show a postoperative AKI incidence of about 30% [12, 17, 41] These discrepancies can be attributed to the fact that these studies defined AKI according to the AKIN or RIFLE criteria [17, 41], which are known to be less sensitive than the KDIGO classification criteria we used [42]. Conversely, Ikehata et al. [12] used KDIGO criteria but they excluded all patients who presented a postoperative infection and AKI was evaluated only during the 7 first postoperative days [12]. In addition, all our patients were systematically admitted to the intermediate care unit where continuous monitoring allows an early and more accurate diagnosis of postoperative complications as compared to standard surgical ward [43]. Along this line, a significant increase in serum creatinine on day 1 was also associated with the occurrence of postoperative AKI, which occurred 4.5 days after surgery. This parameter reflects immediate variations of preoperative serum creatinine and is an indirect indicator of the impact of preoperative renal function on postoperative AKI, as has already been described in this situation [13].

### Intraoperative fluid management and postoperative AKI

An outstanding result of our study is that a restrictive intraoperative vascular filling strategy was independently associated with an increased risk of postoperative AKI in patients undergoing radical cystectomy. With regard to ERAS protocols for major abdominal surgery, it has been shown that a restrictive fluid management strategy may be associated with fewer postoperative complications [44]. However, this approach may carry a high risk of acute renal failure. Indeed, Shin et al. showed in a cohort of 92,094 patients scheduled for non-cardiac surgery under general anesthesia and extracted from a large hospital registry database, that intraoperative fluid volume was associated with a U-shaped curve in which liberal and restrictive volumes were significantly associated with acute kidney injury [45]. Furthermore, moderate restrictive volumes (6–7 mL/kg/h) were consistently associated with optimal postoperative outcomes. This appears to be particularly relevant in patients at high risk of postoperative complications after major abdominal surgery. Indeed, Myles et al. recently showed, in a randomized controlled trial including 3000 high-risk patients for complications during major abdominal surgery, that restrictive fluid regimen (5 ml/kg/h) was associated with a higher rate of acute kidney injury and RRT during the postoperative period [46]. Taken together, these results strongly suggest that a restrictive intraoperative vascular filling strategy may be deleterious in patients at high risk of AKI. Patients undergoing cystectomy represent a high-risk population for postoperative AKI due to pre-existing renal dysfunction related to the extent of preoperative renal obstruction or baseline CKD but also to certain comorbidities such as age or an antihypertensive medication [17]. In this context a too restrictive approach to administration of crystalloids may be associated with an increased risk for AKI [17] whereas this strategy seems to be associated with fewer renal complications after major abdominal surgery in general population [47]. Currently, the intraoperative restrictive fluid management approach is integrated into most ERAS protocols whatever the type of surgery. In a recent retrospective study, the impact of ERAS protocol including intraoperative fluid restriction showed that ERAS protocol increased postoperative AKI incidence in patients undergoing RC, particularly for patients with preoperative CKD [13]. In contrast, in our study, neither the compliance with ERAS protocol nor the period of time during which the ERAS protocol was performed were associated with the occurrence of postoperative AKI. Only a restrictive fluid management in accordance with ERAS protocols (<5ml/kg/h), whatever the period, was strongly and independently associated with AKI. Our results are in line with a recent meta-analysis [48] showing that an intraoperative goal-directed fluid therapy may not be of benefit to all elective patients undergoing major abdominal surgery. This appears to be particularly obvious for patients managed in a preexisting ERAS setting in contrast to those managed in a traditional care setting. Our study suggests that fluid restriction should be used cautiously and not be delivered in all patients undergoing RC.

### Postoperative AKI and outcomes

Another important point in our study is that postoperative AKI is strongly associated with the occurrence or worsening of CKD postoperative period. Our results are consistent with Hannah’s study showing that patients with postoperative AKI demonstrated significantly higher rates of about 30% of CKD following surgery after controlling for baseline renal function. This suggests that postoperative AKI of whatever severity and duration may have consequences for long-term renal function [13]. Indeed, the most marked decline in renal function occurs in the first two years after cystectomy [49]. For this reason, we set the follow-up of these patients at 2 years after cystectomy. An appropriate fluid management strategy using adequate monitoring tools for patients at risk for AKI/CKD undergoing radical cystectomy probably needs to be evaluated. An appropriate fluid management strategy using adequate monitoring tools for patients at risk for AKI/CKD undergoing radical cystectomy probably needs to be evaluated. An important point in our study is that postoperative AKI was not associated with short- or long-term mortality. Overall, the occurrence of postoperative AKI is associated with higher mortality and longer hospital stays whatever the surgery [50]. Concerning RC, AKI is a frequent postoperative complication, which is usually associated with longer hospital stays, higher mortality rates but also long-term impairment of renal function that may lead to the development of chronic kidney disease (CKD) [13]. The lack of impact on mortality, length of stay and readmissions could be explained by the fact that all our patients are systematically admitted to the IMC. In fact, our patients who developed AKI had significantly higher severity scores (SOFA and SAPS II) on admission to IMC than patients who did not develop AKI. Close monitoring of these high-risk patients could improve their prognosis. Indeed, it has been shown that systematic admission of surgical cancer patients to IMC may have beneficial effects on outcomes [6, 43, 51].

Our study presents several limitations. First, its retrospective nature is intrinsically susceptible to induce selection bias, in this situation, we were not be obviously able to have control over many variables. However, all perioperative parameters were collected prospectively with our information system. Second, we cannot totally exclude a time effect on AKI occurrence since urological ERAS protocols were mainly used during the second period of the study. However, the 2 periods during which analyses were performed were not associated with AKI in either univariate or multivariate analysis. Third, we arbitrarily chose to assess the occurrence of AKI up to day 30, partly because the majority of postoperative complications are assessed up to this time in the Dindo-Clavien classification, but also because recent studies assess this endpoint up to day 30 [52]. This is a controversial point, as some have recently recommended defining postoperative AKI as occurring when the existing KDIGO criteria for AKI are met within 7 days of surgery, AKI occurring de novo ≥7 days after surgery can occur in a variety of contexts not necessarily related to the surgery itself [53]. Finally, this study was performed in a single center, this could explain the homogeneity in terms of patients care and might constitute a bias. Thus, these results must therefore be analyzed with caution and cannot be generalized to other centers.

## Conclusion

A restrictive intraoperative vascular filling < 5ml/kg/h was strongly and independently associated with the occurrence of postoperative AKI after RC in cancer bladder patients. In this context, postoperative AKI was also associated with the occurrence or worsening of CKD within the 2 years following RC. A personalized perioperative fluid management strategy needs to be evaluated in these high-risk patients.

## Supporting information

S1 File (DOCX)

## Abbreviations

AKI: Acute Kidney Injury
ASA: American Society of Anesthesiologist
BMI: Body Mass Index
CKD: chronic kidney disease
Delta SCr: Difference between serum creatinine level
ERAS: Early Recovered After Surgery
GEMCis: Gemcitabin-Cisplatin
GFR: Glomerular Filtration Rate
GFR: Glomerular Filtration Rate
KDIGO: Kidney Disease Improving Global Outcomes
MTX: Methotrexate
MVAC: Methotrexate-Vinblastine-Doxorubicin-Cisplatin
NSAIDs: Non-Steroidal Anti-Inflammatory Drugs
OR: odds ratio
RAAS: Renin–angiotensin–aldosterone system
RC: radical cystectomy
RRT: renal replacement therapy
SAPS: Simplified Acute Physiology Score
Scr: Serum Creatinine level
sHR: sub hazard ratio
SOFA: Sequential Organ Failure Assessment

## References

[1] Williams SB, Cumberbatch MGK, Kamat AM, Jubber I, Kerr PS, McGrath JS, et al. Reporting Radical Cystectomy Outcomes Following Implementation of Enhanced Recovery After Surgery Protocols: A Systematic Review and Individual Patient Data Meta-analysis. Eur Urol. 2020;78: 719–730. doi: 10.1016/j.eururo.2020.06.039 32624275

[2] Ni X, Jia D, Chen Y, Wang L, Suo J. Is the Enhanced Recovery After Surgery (ERAS) Program Effective and Safe in Laparoscopic Colorectal Cancer Surgery? A Meta-Analysis of Randomized Controlled Trials. J Gastrointest Surg Off J Soc Surg Aliment Tract. 2019;23: 1502–1512. doi: 10.1007/s11605-019-04170-8 30859422

[3] Melnyk M, Casey RG, Black P, Koupparis AJ. Enhanced recovery after surgery (ERAS) protocols: Time to change practice? Can Urol Assoc J J Assoc Urol Can. 2011;5: 342–348. doi: 10.5489/cuaj.11002 22031616 PMC3202008

[4] Lambaudie E, Mathis J, Zemmour C, Jauffret-Fara C, Mikhael ET, Pouliquen C, et al. Prediction of early discharge after gynaecological oncology surgery within ERAS. Surg Endosc. 2020;34: 1985–1993. doi: 10.1007/s00464-019-06974-w 31309314

[5] Meillat H, Brun C, Zemmour C, de Chaisemartin C, Turrini O, Faucher M, et al. Laparoscopy is not enough: full ERAS compliance is the key to improvement of short-term outcomes after colectomy for cancer. Surg Endosc. 2020;34: 2067–2075. doi: 10.1007/s00464-019-06987-5 31385073

[6] Canac J, Faucher M, Depeyre F, Tourret M, Tezier M, Cambon S, et al. Factors Associated with 1-Year Mortality in Elderly Patients (Age ≥ 80 Years) with Cancer Undergoing Major Abdominal Surgery: A Retrospective Cohort Study. Ann Surg Oncol. 2023;30: 8083–8093. doi: 10.1245/s10434-023-14365-8 37814178

[7] Wuethrich PY, Burkhard FC, Thalmann GN, Stueber F, Studer UE. Restrictive deferred hydration combined with preemptive norepinephrine infusion during radical cystectomy reduces postoperative complications and hospitalization time: a randomized clinical trial. Anesthesiology. 2014;120: 365–377. doi: 10.1097/ALN.0b013e3182a44440 23887199

[8] Egal M, de Geus HRH, van Bommel J, Groeneveld ABJ. Targeting oliguria reversal in perioperative restrictive fluid management does not influence the occurrence of renal dysfunction: A systematic review and meta-analysis. Eur J Anaesthesiol. 2016;33: 425–435. doi: 10.1097/EJA.0000000000000416 26840829

[9] Sear JW. Kidney dysfunction in the postoperative period. Br J Anaesth. 2005;95: 20–32. doi: 10.1093/bja/aei018 15531622

[10] Hautmann RE, de Petriconi RC, Pfeiffer C, Volkmer BG. Radical Cystectomy for Urothelial Carcinoma of the Bladder Without Neoadjuvant or Adjuvant Therapy: Long-Term Results in 1100 Patients. Eur Urol. 2012;61: 1039–1047. doi: 10.1016/j.eururo.2012.02.028 22381169

[11] Osman Y, Harraz AM, El-Halwagy S, Laymon M, Mosbah A, Abol-Enein H, et al. Acute kidney injury following radical cystectomy and urinary diversion: predictors and associated morbidity. Int Braz J Urol. 2018;44: 726–733. doi: 10.1590/S1677-5538.IBJU.2017.0283 29757568 PMC6092670

[12] Ikehata Y, Tanaka T, Ichihara K, Kobayashi K, Kitamura H, Takahashi S, et al. Incidence and risk factors for acute kidney injury after radical cystectomy. Int J Urol. 2016;23: 558–563. doi: 10.1111/iju.13104 27168129

[13] Hanna PT, Peterson M, Albersheim J, Drawz P, Zabell J, Konety B, et al. Acute Kidney Injury following Enhanced Recovery after Surgery in Patients Undergoing Radical Cystectomy. J Urol. 2020;204: 982–988. doi: 10.1097/JU.0000000000001153 32469268

[14] Joung K-W, Choi S-S, Kong Y-G, Yu J, Lim J, Hwang J-H, et al. Incidence and Risk Factors of Acute Kidney Injury after Radical Cystectomy: Importance of Preoperative Serum Uric Acid Level. Int J Med Sci. 2015;12: 599–604. doi: 10.7150/ijms.12106 26283877 PMC4532964

[15] Lafrance J-P, Miller DR. Acute Kidney Injury Associates with Increased Long-Term Mortality. J Am Soc Nephrol JASN. 2010;21: 345–352. doi: 10.1681/ASN.2009060636 20019168 PMC2834549

[16] O’Connor ME, Kirwan CJ, Pearse RM, Prowle JR. Incidence and associations of acute kidney injury after major abdominal surgery. Intensive Care Med. 2016;42: 521–530. doi: 10.1007/s00134-015-4157-7 26602784

[17] Furrer MA, Schneider MP, Löffel LM, Burkhard FC, Wuethrich PY. Impact of intra-operative fluid and noradrenaline administration on early postoperative renal function after cystectomy and urinary diversion: A retrospective observational cohort study. Eur J Anaesthesiol. 2018;35: 641–649. doi: 10.1097/EJA.0000000000000808 29652680

[18] Macedo E, Zanetta DMT, Abdulkader RCRM. Long-term follow-up of patients after acute kidney injury: patterns of renal functional recovery. PloS One. 2012;7: e36388. doi: 10.1371/journal.pone.0036388 22574153 PMC3344858

[19] Malavaud B, Malavaud B, Vaessen C, Vaessen C, Mouzin M, Mouzin M, et al. Complications for Radical Cystectomy. Eur Urol. 2001;39: 79–84. 11173943 10.1159/000052416

[20] Jones J, Holmen J, De Graauw J, Jovanovich A, Thornton S, Chonchol M. Association of complete recovery from acute kidney injury with incident CKD stage 3 and all-cause mortality. Am J Kidney Dis Off J Natl Kidney Found. 2012;60: 402–408. doi: 10.1053/j.ajkd.2012.03.014 22541737 PMC3422603

[21] Nishikawa M, Miyake H, Yamashita M, Inoue T, Fujisawa M. Long-term changes in renal function outcomes following radical cystectomy and urinary diversion. Int J Clin Oncol. 2014;19: 1105–1111. doi: 10.1007/s10147-014-0661-y 24445559

[22] Schmidt B, Velaer KN, Thomas I-C, Ganesan C, Song S, Pao AC, et al. Renal Morbidity Following Radical Cystectomy in Patients with Bladder Cancer. Eur Urol Open Sci. 2022;35: 29–36. doi: 10.1016/j.euros.2021.11.001 35024629 PMC8738897

[23] Nguyen C, Ghodoussipour S, Winter M, Cacciamani G, Ahmadi H, Djaladat H, et al. Chronic kidney disease and radical cystectomy for bladder cancer: perioperative and oncologic outcomes in 1,214 patients. Urol Oncol. 2022;40: 381.e9–381.e16. doi: 10.1016/j.urolonc.2022.04.010 35599109

[24] Rouprêt M, Neuzillet Y, Pignot G, Compérat E, Audenet F, Houédé N, et al. RETRACTED: Recommandations françaises du Comité de Cancérologie de l’AFU—Actualisation 2018–2020: tumeurs de la vessie. Prog En Urol. 2018;28: S46–S78. 10.1016/j.purol.2018.07.283 30366708

[25] Poinas G, Blache JL, Kassab-Chahmi D, Evrard PL, Artus PM, Alfonsi P, et al. Version courte des recommandations de la récupération ameliorée après chirurgie (RAAC) pour la cystectomie: mesures techniques. Prog En Urol. 2019;29: 63–75. 10.1016/j.purol.2018.12.002 30635149

[26] Futier E, Constantin J-M, Paugam-Burtz C, Pascal J, Eurin M, Neuschwander A, et al. A trial of intraoperative low-tidal-volume ventilation in abdominal surgery. N Engl J Med. 2013;369: 428–437. doi: 10.1056/NEJMoa1301082 23902482

[27] Apfel CC, Läärä E, Koivuranta M, Greim CA, Roewer N. A simplified risk score for predicting postoperative nausea and vomiting: conclusions from cross-validations between two centers. Anesthesiology. 1999;91: 693–700. doi: 10.1097/00000542-199909000-00022 10485781

[28] Mokart D, Giaoui E, Barbier L, Lambert J, Sannini A, Chow-Chine L, et al. Postoperative sepsis in cancer patients undergoing major elective digestive surgery is associated with increased long-term mortality. J Crit Care. 2016;31: 48–53. doi: 10.1016/j.jcrc.2015.10.001 26507291

[29] Levey AS, Coresh J. Chronic kidney disease. The Lancet. 2012;379: 165–180. doi: 10.1016/S0140-6736(11)60178-5 21840587

[30] Charlson ME, Pompei P, Ales KL, MacKenzie CR. A new method of classifying prognostic comorbidity in longitudinal studies: Development and validation. J Chronic Dis. 1987;40: 373–383. doi: 10.1016/0021-9681(87)90171-8 3558716

[31] The Clinical Evaluation of Chemotherapeutic Agents in Cancer–ScienceOpen. [cited 5 May 2021]. https://www.scienceopen.com/document?vid=b6149047-7b97-4b0b-bfef-995cb93d5174

[32] Keats AS. The ASA classification of physical status-A recapitulation. Anesthesiology. oct 1978;49(4):233‑6. doi: 10.1097/00000542-197810000-00001 697075

[33] Le Gall JR, Lemeshow S, Saulnier F. A new Simplified Acute Physiology Score (SAPS II) based on a European/North American multicenter study. JAMA. 1993;270: 2957–2963. doi: 10.1001/jama.270.24.2957 8254858

[34] Dindo D, Demartines N, Clavien P-A. Classification of Surgical Complications: A New Proposal With Evaluation in a Cohort of 6336 Patients and Results of a Survey. Ann Surg. 2004;240: 205–213. doi: 10.1097/01.sla.0000133083.54934.ae 15273542 PMC1360123

[35] Singer M, Deutschman CS, Seymour CW, Shankar-Hari M, Annane D, Bauer M, et al. The Third International Consensus Definitions for Sepsis and Septic Shock (Sepsis-3). JAMA. 2016;315: 801–810. doi: 10.1001/jama.2016.0287 26903338 PMC4968574

[36] Khwaja A. KDIGO clinical practice guidelines for acute kidney injury. Nephron Clin Pract. 2012;120: c179–184. doi: 10.1159/000339789 22890468

[37] National Kidney Foundation. K/DOQI clinical practice guidelines for chronic kidney disease: evaluation, classification, and stratification. Am J Kidney Dis Off J Natl Kidney Found. 2002;39: S1–266. 11904577

[38] Levey AS, Coresh J. Chronic kidney disease. The Lancet. 2012;379: 165–180. doi: 10.1016/S0140-6736(11)60178-5 21840587

[39] Fine JP, Gray RJ. A Proportional Hazards Model for the Subdistribution of a Competing Risk. J Am Stat Assoc. 1999;94: 496. doi: 10.2307/2670170

[40] Gray RJ. A Class of K-Sample Tests for Comparing the Cumulative Incidence of a Competing Risk. Ann Stat. 1988;16. doi: 10.1214/aos/1176350951

[41] Kwon T, Jeong IG, Lee C, You D, Hong B, Hong JH, et al. Acute Kidney Injury After Radical Cystectomy for Bladder Cancer is Associated with Chronic Kidney Disease and Mortality. Ann Surg Oncol. 2016;23: 686–693. doi: 10.1245/s10434-015-4886-4 26442922

[42] Luo X, Jiang L, Du B, Wen Y, Wang M, Xi X, et al. A comparison of different diagnostic criteria of acute kidney injury in critically ill patients. Crit Care Lond Engl. 2014;18: R144. doi: 10.1186/cc13977 25005361 PMC4227114

[43] Mokart D, Giaoui E, Barbier L, Lambert J, Sannini A, Chow-Chine L, et al. Postoperative sepsis in cancer patients undergoing major elective digestive surgery is associated with increased long-term mortality. J Crit Care. 2016;31: 48–53. doi: 10.1016/j.jcrc.2015.10.001 26507291

[44] Benes J, Giglio M, Brienza N, Michard F. The effects of goal-directed fluid therapy based on dynamic parameters on post-surgical outcome: a meta-analysis of randomized controlled trials. Crit Care Lond Engl. 2014;18: 584. doi: 10.1186/s13054-014-0584-z 25348900 PMC4234857

[45] Shin CH, Long DR, McLean D, Grabitz SD, Ladha K, Timm FP, et al. Effects of Intraoperative Fluid Management on Postoperative Outcomes: A Hospital Registry Study. Ann Surg. 2018;267: 1084–1092. doi: 10.1097/SLA.0000000000002220 28288059

[46] Myles PS, Bellomo R, Corcoran T, Forbes A, Peyton P, Story D, et al. Restrictive versus Liberal Fluid Therapy for Major Abdominal Surgery. N Engl J Med. 2018;378: 2263–2274. doi: 10.1056/NEJMoa1801601 29742967

[47] Corcoran T, Emma Joy Rhodes J, Clarke S, Myles PS, Ho KM. Perioperative Fluid Management Strategies in Major Surgery: A Stratified Meta-Analysis. Anesth Analg. 2012;114: 640–651. doi: 10.1213/ANE.0b013e318240d6eb 22253274

[48] Rollins KE, Lobo DN. Intraoperative Goal-directed Fluid Therapy in Elective Major Abdominal Surgery: A Meta-analysis of Randomized Controlled Trials. Ann Surg. 2016;263: 465–476. doi: 10.1097/SLA.0000000000001366 26445470 PMC4741406

[49] Makino K, Nakagawa T, Kanatani A, Kawai T, Taguchi S, Otsuka M, et al. Biphasic decline in renal function after radical cystectomy with urinary diversion. Int J Clin Oncol. 2017;22: 359–365. doi: 10.1007/s10147-016-1053-2 27747456

[50] Zarbock A, Weiss R, Albert F, Rutledge K, Kellum JA, Bellomo R, et al. Epidemiology of surgery associated acute kidney injury (EPIS-AKI): a prospective international observational multi-center clinical study. Intensive Care Med. 2023;49: 1441–1455. doi: 10.1007/s00134-023-07169-7 37505258 PMC10709241

[51] POSE-Study group. Peri-interventional outcome study in the elderly in Europe: A 30-day prospective cohort study. Eur J Anaesthesiol. 2022;39: 198–209. doi: 10.1097/EJA.0000000000001639 34799496 PMC8815832

[52] Lone Z, Campbell RA, Corrigan D, Ramkumar R, Hegde P, Rahmy A, et al. Ability of the surgical Apgar score to predict acute kidney injury following radical cystectomy. Urol Oncol Semin Orig Investig. 2022;40: 194.e1–194.e6. doi: 10.1016/j.urolonc.2021.09.006 34654645

[53] Prowle JR, Forni LG, Bell M, Chew MS, Edwards M, Grams ME, et al. Postoperative acute kidney injury in adult non-cardiac surgery: joint consensus report of the Acute Disease Quality Initiative and PeriOperative Quality Initiative. Nat Rev Nephrol. 2021;17: 605–618. doi: 10.1038/s41581-021-00418-2 33976395 PMC8367817

