# Supplementary material for: Risk factors for postoperative acute kidney injury after radical cystectomy for bladder cancer in the era of ERAS protocols: A retrospective observational study
Source: PLoS One. 2024 Oct 15;19(10):e0309549. doi: 10.1371/journal.pone.0309549 (PMC11478916; doi:10.1371/journal.pone.0309549)
Supplement: S1 File — (DOCX) [file pone.0309549.s001.docx]

**Supplementary Files**

1. **Supplementary file 1: Summary of ERAS protocol**

**Days to weeks prior to surgery**

• Shared decision making and surgical consent

• Stoma nurse review and advice

• Dietician review and advice

• Physiotherapist prehabilitation advice

• Patient “mentors” through local bladder cancer patient forum

• Cardiopulmonary exercise testing

• Medical optimization

• Cessation of smoking

**Day prior to surgery**

• Admission to hospital evening prior to facilitate administration of carbohydrate pre-load, stoma site marking and phosphate enemas

• Starved of solid food 6 hours prior to surgery, and clear fluids stopped 2 hours prior

• Avoidance of mechanical bowel preparation

Intraoperative measures

**Anesthesia**

• Standardized anesthesia protocol

• Intrathecal analgesia

• Avoidance of long acting pre-medication

• Antisialagogue to reduce secretions

• Antimicrobial prophylaxis

• Intraoperative normothermia

• Avoid excess intravenous fluids whilst steep Trendelenburg and clamped ureters

• Close monitoring of potassium levels

• Ventilator strategy to minimise airway pressures

• Endotracheal tube tape to avoid venous congestion

• BIS monitoring to avoid excessive anaesthesia

• Avoidance of nasogastric tubes

• Aid lung recruitment and reduce cerebral oedema with sitting patient and slow wake up postoperatively

**Surgical**

• Robot assisted

• Valve less insufflation system to stabilise pneumoperitoneum

• Small incisions

**Postoperative measures**

**Anaesthesia**

• Individualized fluid therapy with goal directed fluid therapy

• Early disconnection of intravenous fluids after stroke volume optimization

• Encouraging oral fluids and normal diet

• Nutritional support

• Thromboprophylaxis

**Surgical**

• Regular oral analgesics

• Regular physiotherapy including deep breathing and incentive spirometry

• Chewing gum to reduce post-operative ileus

• Early removal of pelvic drain

• Early teaching of stoma care

1. **Supplementary File 2: Anaesthesia and analgesia protocols**

In all patients, induction of general anesthesia (GA) was performed using propofol (3-5 mg/kg) and remifentanil (target-controlled infusion and halogenated inhalation anesthetic (desflurane or sevoflurane in an oxygen/air mixture) with bispectral index monitoring for the maintenance of GA. Orotracheal intubation was used for airway management after administration of neuromuscular blockade with cisatracurium (0,15 mg/kg). Repeated bolus injections of cisatracurium and continuous administration of remifentanil were given as necessary throughout the operation

1. **Supplementary File 3: Variables used for evaluating compliance to ERAS protocol on patients before and from 01/2018**

|  |
| --- |
|  |
| Pre operative ERAS items |
| Pre-operative counselling and education |
| Pre operative medical optimization |
| Nutritional evaluation |
| Pre operative respiratory preparation |
| Pre oprerative management of aneamia |
| No sedative premedication |
| Preoperative carbohydrate loading |
| Selective bowel preparation |
| Peri operative ERAS items |
| Antibiotic prophylaxis |
| Postoperative nausea and vomiting prophylaxis |
| Glucocorticoid loading |
| Opioid sparing strategy* |
| Short acting opioids |
| Haemodynamics monitoring |
| Restrictive intraoperative vascular filling** |
| Protective ventilation*** |
| Robot assisted surgery |
| Maintaining normothermia |
| Neuromuscular blockers monitoring |
| Post operative ERAS items |
| Antithrombotic prophylaxis |
| Ileus prevention (early mobilisation and chewing gum) |
| Avoidance of abdominal drain**** |
| Post operative physiotherapy |
| Early mobilisation |

* We considered opioid sparing strategy when multimodal analgesia in addition to morphine PCA was used. **Intraoperative vascular filling < 5ml/kg/h. *** Ventilation was protective when it follows international guidelines: tidal volume between 6 and 8 ml.kg-1, PEEP 5 cm H2O and Pplat<30 cm H2O). ****Abdominal drainage was considered correct when none or one drain was let.

1. **Supplementary File 4**

- For the logistic regression model, it is usual to include one parameter for every 5 to 10 events. We decided to include 1 parameter for every 7 events. Given that 51 patients presented with postoperative AKI, we selected 7 perioperative parameters that were significant in univariate analysis and of clinical relevance to the clinician. These parameters were: intraoperative restrictive vascular filling, postoperative sepsis, female sex, SOFA score at day 1, delta serum creatinine at day 1, duration of surgery and laparotomy during surgery. It is important to note that when all the significant parameters are included in the logistic regression model without any a priori selection, the results of the multivariate analysis remain identical.
- Fine & Gray's approach was different, but ultimately similar. We wanted to evaluate whether the occurrence of a postoperative AKI could have an impact on the occurrence of CKD or the deterioration of a pre-existing CKD, independently of certain confounding factors that we had pre-determined and that were already described in the results section (MVAC, GemCis, preoperative CKD, Charlson comorbidity index, ASA score, intraoperative restrictive fluid management, intraoperative vasopressors, ERAS protocol, robot assisted surgery, SOFA score at day 1,serum potassium level at day 1, serum potassium level at day 3, SAPS II score, postoperative complications = Clavien-Dindo stages and postoperative RRT). Of these parameters, only 3 were significant in univariate analysis and were included in the Fine & Gray model. The results of the univariate and multivariate analysis are now described in the table in Supplementary File 5.

1. **Supplementary file 5 : Risk for occurrence or worsening of a CKD within the 2 years**

| **Univariate analysis Multivariate analysis** |
| --- |
| **p value sHR (95%CI) p value sH (95%CI)** |

| Restrictive IV filling < 5 ml/kg/h | 0.149 | 1.67 | (0.83-3.34) |  |  |  |
| --- | --- | --- | --- | --- | --- | --- |
| No complication | reference |  |  |  |  |  |
| Clavien-Dindo I | 0.055 | 4.36 | (0.97-19.55) |  |  |  |
| Clavien-Dindo II | 0.436 | 1.55 | (0.51-4.67) |  |  |  |
| Clavien-Dindo III | 0.318 | 1.91 | (0.54-6.76) |  |  |  |
| Clavien-Dindo IV | 0.031 | 3.75 | (1.13-12.46) |  |  |  |
| Postoperative AKI | 0.019 | 2.47 | (1.16-5.26) | 0.037 | 2.25 | (1.05-4.81) |
| ERAS protocols | 0.633 | 1.19 | (0.58-2.44) |  |  |  |
| Robot surgery | 0.372 | 1.37 | (0.69-2.71) |  |  |  |
| SOFA day 1 | 0.744 | 1.04 | (0.84-1.28) |  |  |  |
| SAPS II | 0.435 | 1.01 | (0.98-1.05) |  |  |  |
| MVAC | 0.820 | 1.08 | (0.55-2.14) |  |  |  |
| GEMCIS | 0.758 | 0.85 | (0.30-2.41) |  |  |  |
| RRT | 0.177 | 2.68 | (0.64-11.21) |  |  |  |
| Serum potassium level day 1 | 0.813 | 1.09 | (0.54-2.21) |  |  |  |
| Serum potassium level day 3 | 0.010 | 3.67 | (1.36-9.94) | 0.049 | 2.04 | (1.00-4.16) |
| Intraoperative norepinephrine | 0.934 | 0.96 | (0.34-2-71) |  |  |  |
| Charlson score | 0.224 | 1.14 | (0.92-1.41) |  |  |  |
| ASA score >2 | 0.564 | 1.22 | (0.63-2.36) |  |  |  |
| GFR< 60 ml/min/1,73m^2^ | 0.219 | 1.65 | (0.74-2.85) |  |  |  |
